# Imaging Features of Post Main Hepatectomy Complications: The Radiologist Challenging

**DOI:** 10.3390/diagnostics12061323

**Published:** 2022-05-26

**Authors:** Carmen Cutolo, Federica De Muzio, Roberta Fusco, Igino Simonetti, Andrea Belli, Renato Patrone, Francesca Grassi, Federica Dell’Aversana, Vincenzo Pilone, Antonella Petrillo, Francesco Izzo, Vincenza Granata

**Affiliations:** 1 Department of Medicine, Surgery and Dentistry, University of Salerno, 84084 Fisciano, Italy; carmencutolo@hotmail.it (C.C.); vpilone@unisa.it (V.P.); 2 Department of Medicine and Health Sciences V. Tiberio, University of Molise, 86100 Campobasso, Italy; demuziofederica@gmail.com; 3 Medical Oncology Division, Igea SpA, 80013 Naples, Italy; r.fusco@igeamedical.com; 4 Radiology Division, Istituto Nazionale Tumori—IRCCS—Fondazione G. Pascale, Via Mariano Semmola, 80131 Naples, Italy; igino.simonetti@istitutotumori.na.it (I.S.); a.petrillo@istitutotumori.na.it (A.P.); 5 Hepatobiliary Surgical Oncology Division, Istituto Nazionale Tumori—IRCCS—Fondazione G. Pascale, Via Mariano Semmola, 80131 Naples, Italy; a.belli@istitutotumori.na.it (A.B.); dott.patrone@gmail.com (R.P.); f.izzo@istitutotumori.na.it (F.I.); 6 Division of Radiology, Università degli Studi della Campania Luigi Vanvitelli, 81100 Naples, Italy; francesca.grassi1@studenti.unicampania.it (F.G.); federica.dellaversana@studenti.unicampania.it (F.D.); 7 Italian Society of Medical and Interventional Radiology (SIRM), SIRM Foundation, Via della Signora 2, 20122 Milan, Italy

**Keywords:** hepatectomy, postoperative complications, radiologists

## Abstract

In the recent years, the number of liver resections has seen an impressive growth. Usually, hepatic resections remain the treatment of various liver diseases, such as malignant tumors, benign tumors, hydatid disease, and abscesses. Despite technical advancements and tremendous experience in the field of liver resection of specialized centers, there are moderately high rates of postoperative morbidity and mortality, especially in high-risk and older patient populations. Although ultrasonography is usually the first-line imaging examination for postoperative complications, Computed Tomography (CT) is the imaging tool of choice in emergency settings due to its capability to assess the whole body in a few seconds and detect all possible complications. Magnetic resonance cholangiopancreatography (MRCP) is the imaging modality of choice for delineating early postoperative bile duct injuries and ischemic cholangitis that may arise in the late postoperative phase. Moreover, both MDCT and MRCP can precisely detect tumor recurrence. Consequently, radiologists should have knowledge of these surgical procedures for better comprehension of postoperative changes and recognition of the radiological features of various postoperative complications.

## 1. Introduction

Liver resection is still the most efficient treatment of primary liver malignancies, including hepatocellular carcinoma (HCC) and cholangiocarcinoma (CCA), and in metastatic disease, such as colorectal liver metastases [1,2,3,4,5,6,7,8,9]. According to the increase in occurrence of these primary and metastatic cancers, the number of hepatic resections is globally rising, and it doubled in the USA from 1988 to 2000 [10,11]. The advancement in patient selection and the innovative surgical techniques have decreased the risk of mortality from 20% to 1–5% [12,13]. In spite of this, morbidity rates even now vary from 20 to 56%, depending on the patient characteristics, such as tumor size and localization, and multidisciplinary team expertise and available technologies [12,13,14,15]. As reported by Benzoni et al., major hepatectomies, Pringle maneuver protracted more than 20 min and blood transfusions greater than 600 mL were associated with significant increases in complications. Furthermore, types B and C of the Child–Pugh classification and histopathologic grading are correlated with higher complications in patients with HCC [16]. Sadamori et al. [17] reported a prominently higher frequency of bile leakage (12.8% overall) and organ/space surgical site infections (8.6% overall) in patients undergoing repeat hepatectomy and prolonged surgery.

To summarize, increasing age with significant related comorbidities, extended resections, and iterative hepatectomies are all risk factors for the development of postoperative complications. Moreover, for colorectal metastasis, if, on one hand, a preoperative chemotherapy regimen converted the lesion previously believed unresectable to resectable, on the other hand, after chemotherapy, the liver is more subject to steatosis and steatohepatitis with greater frequency of postoperative complications [18,19,20,21,22].

Radiology plays a key role in the early discovery of postoperative complications. In fact, recent progress in diagnostic imaging modalities such as computed tomography (CT) [23,24,25,26,27,28,29,30,31,32,33,34] or magnetic resonance imaging (MRI) [35,36,37,38,39,40,41,42,43,44,45,46,47,48] with cholangiopancreatography have enabled an exact assessment of the postoperative morphological changes of the remaining liver, as well as a determination and an evaluation of postoperative complications [49,50,51,52].

The aim of this work is to summarize the main posthepatectomy complications and their radiological features.

## 2. Type of Resections

When discussing potential complications after liver resections, it is fundamental to specify the extent of the resection.

Liver resections (hepatectomies) can be categorized into anatomical and nonanatomical resections.

Anatomical resections consist of the removal of contiguous functional liver segments, while nonanatomic liver resections consist of the removal of the tumor with a margin of at least 1 cm without regard to segmental, sectional, or lobar anatomy [53,54,55]. Despite the number of segments removed, it is not enough to represent the complexity of a liver resection [56]; major hepatectomies are commonly defined as the resection of three segments in the left liver and four segments in the right liver.

The Terminology Committee of the International Hepato-Pancreato-Biliary Association defined a standardized nomenclature of anatomical resections in 2000 [57]. A right hepatectomy includes the removal of segments 5, 6, 7, and 8. It consists of the removal of all hepatic parenchyma to the right of the middle hepatic vein. An extended right hepatectomy (or right trisectionectomy) includes the additional resection of segment 4 (left medial section).

A left hepatectomy includes the removal of segments 2, 3, and 4 and consists of the removal of all hepatic parenchyma to the left of the middle hepatic vein. An extended left hepatectomy (or left trisectionectomy) involves the additional resection of segments 5 and 8 (right anterior section) [57]. Sectionectomies are defined by the type of section removed. A right anterior sectionectomy consists of the removal of the right anterior section—segments 5 and 8. A monosegmentectomy consists of the removal of a single segment, while a bisegmentectomy involves the resection of two contiguous segments [57]. Several studies demonstrated comparable morbidity in different types of liver resections, whereas others have shown significant differences in major hepatectomies. Zimmitti et al. [58] analyzed the incidence rates of postoperative complications in increasingly complex liver resections. They showed that, except for biliary leaks, the percentage of complications did not increase as the complexity of the operation increased. Li et al. [59] demonstrated that a major hepatectomy was related to greater rates of infectious (organ/space, superficial skin infections, pneumonia, sepsis, and septic shock), pulmonary (unplanned reintubation and prolonged ventilator support), renal (progressive renal insufficiency and acute renal failure), and hematologic (bleeding within 72 h and deep venous thrombosis [DVT]) complications, when compared with minor hepatectomies [59].

A factor that should be considered is the pathology of the underlying liver. At least 80% of HCC patients will develop hepatic fibrosis or cirrhosis [60,61,62,63,64,65,66,67,68].

Accordingly, the remnant liver is already damaged and perhaps more vulnerable to further injury.

## 3. Complications

Complications, defined as any unexpected modification from a procedural course, and adverse events, described as any existent or potential injury connected with the treatment, could occur either during the procedure or after the procedure [69,70,71,72,73].

A major complication is an event that results in substantial morbidity and injury, allowing an increase in the level of care or resulting in hospital admission or a protracted hospital stay. Circumstances that are different from this condition are described as minor complications [69,70,71,72,73].

Postprocedural complications are a frequent occurrence after hepatic resections and differ based on the type of surgical procedure, the type of intervention on the biliary ducts and vascular structures, the grade and histological type of the treated tumor, and the existence of an underlying chronic disease [74,75,76,77,78]. According to the time of onset, postoperative complications can be defined as early and late complications. Fluid collection, vascular thrombosis, vascular or biliary duct damage, and diaphragmatic injuries are the most frequent early postoperative complications [74,75,76,77,78].

The most feared long-term complication is undoubtedly disease recurrence, while ischaemic cholangitis is a mild to severe late complication that could manifest months or even years after the procedure [79,80,81,82,83].

Complications should be assessed according to the following classification systems: (a) Common Terminology Criteria for Adverse Events standards, (b) Clavien–Dindo classification, (c) Society of Interventional Radiology classification, and (d) Cardiovascular and Interventional Radiological Society of Europe Quality Assurance Document and Standards for Classification of Complications [59]; complications should be classified constantly according to severity and time of incidence (e.g., intraprocedural, postprocedural, or late) [74,75,76,77,78].

Various imaging techniques could be used, alone or in association, to successfully assess patients after liver resection. The imaging techniques most commonly used in the detection and characterization of complications are Ultrasound (US) [84,85,86,87] and contrast-enhanced CT (CECT) [88,89,90,91,92,93,94,95,96,97,98,99,100,101].

In addition, Magnetic Resonance Imaging (MRI) is very important in the evaluation of late postoperative complications. In particular, MRI is fundamental in the early characterization of disease recurrence [102,103,104,105,106,107,108,109,110,111,112,113].

### 3.1. Early Postoperative Complications

#### 3.1.1. Fluid Collection

**Figure 1:**
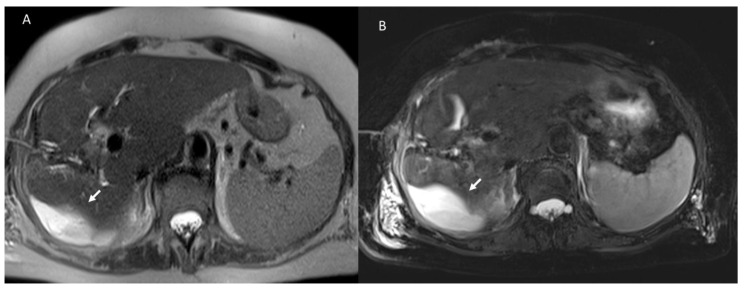
Postsurgical biloma assessed with MRI at 1 week post resection of VIII segment for liver metastasis. The biloma (arrow) appears hyperintense in T2 (**A**,**B**) sequences of MRI study.

**Figure 2:**
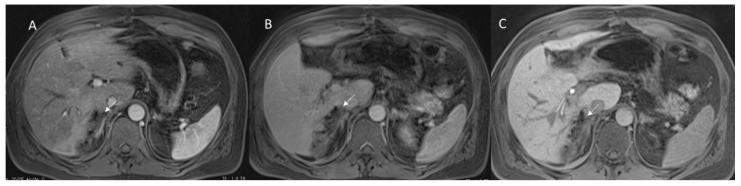
Hepatic abscess in resected cholangiocarcinoma on VI hepatic segment, evaluated with MRI. Arrow shows air artifacts within the collection and hyperenhancement of hepatic parenchymal in arterial phase (**A**) of contrast study that disappears in portal (**B**) and hepatobiliary (**C**) phase of contrast study.

**Figure 3:**
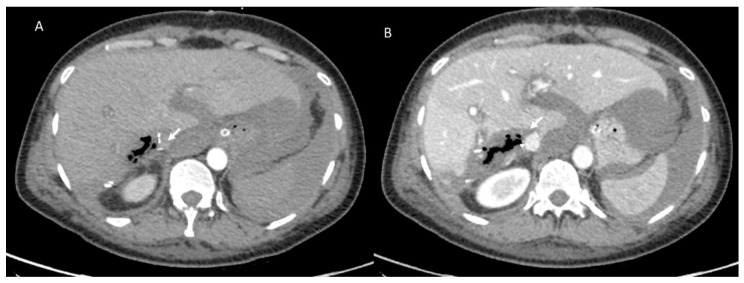
Hepatic abscess in resected hepatocellular carcinoma on VI hepatic segment, evaluated with CT. Arrow shows air artifacts within the collection in arterial (**A**) and portal (**B**) phase of contrast study.

Postsurgical fluid collection could be divided according to composition into hematomas (50%), bilomas (25%) (Figure 1), and abscesses (25%) (Figure 2 and Figure 3) [114]. Collection usually tends to localize along the resection margins that should be carefully investigated during both US and CT examinations [115].

A biconvex or growing intraparenchymal areas, heterogeneous and echogenic on US or with a superfluid density value (between 50 and 60 HU) on unenhanced CT, are strongly suggestive of a hematoma [115]. The suspicion should be confirmed after the administration of a contrast medium agent since the hematoma does not show any contrast enhancement [115].

Biloma could be defined as an encapsulated store of bile outside the biliary tree and within the abdominal cavity [116]. It is more homogeneous than hematomas, with density values much closer to that of water [116]. On US, bilomas appear as simple cyst-like collections, compared to the greater echogenicity of hematomas. In the case of overinfection, the mass tends to appear more structured with a mixed content of cellular debris and bile [115].

**Figure 4:**
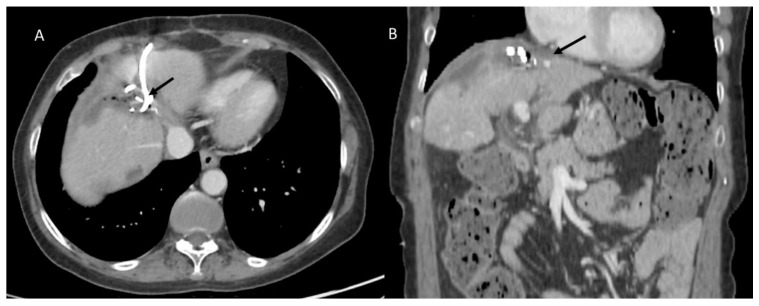
CT-guided hepatic infected biloma drainage (arrows) and postprocedure assessment in portal phase of contrast study in axial (**A**) and coronal (**B**) plane.

The presence of air artifacts detected within the collection and the absence of central perfusion on color Doppler examination in patients with fever and a decline in physical conditions suggest an abscess formation [115]. CT usually confirms the diagnosis with the typical findings of a central hypodense core of fluid material surrounded by a hyperdense rim and a hypodense outer ring as a double target appearance [115]. Percutaneous drainage should be considered in the case of infected collections (abscesses and bilomas) (Figure 4) [117,118,119], when a worsening of laboratory and clinical parameters occurs despite antibiotic therapy.

US is the first-level imaging method in the study of fluid collection, allowing for the definition of the location, dimension, and composition of the lesion, and it could guide the possible drainage. CECT should be performed in doubtful cases. Thanks to its spatial resolution and the possibility to conduct multiplanar reconstructions (MPR), CT allows to evaluate not only complex collections defining boundaries with adjacent structures, but also to reveal possible associated complications [115]. A multiphasic CT protocol should comprise an unenhanced phase that easily detects one hematoma store, an arterial phase to intercept any source of bleeding, and a portal phase that allows to identify hepatic abscesses [115,120,121,122].

#### 3.1.2. Posthepatectomy Hemorrhage

Posthepatectomy hemorrhage (PHH) is a major complication, which can substantially increase morbidity and mortality rates, with a described incidence of 1–8% [123]. In recent years, the International Study Group of Liver Surgery (ISGLS) has suggested a novel definition and staging of PHH with the aim of obtaining a standardized report of complications [124]. According to these guidelines, PHH is defined as a decrease in hemoglobin level >3 g/dL compared to the postoperative baseline level (i.e., hemoglobin level immediately after surgery) and has three grades of severity (A-B-C), depending on the therapeutic strategy needed. In particular, a grade A hemorrhage could be controlled with minimal transfusion, while a grade B may need up to two transfusions in combination with medical anticoagulation therapy and/or the administration of procoagulant agents. Finally, grade C corresponds to a life-threatening situation that requires radiological interventional treatment (such as embolization) or open surgery to manage the bleeding [124].

Currently, the known bleeding causes are: *(a)* bleeding from the surfaces of the remnant liver for arterial branch section or congestion of the hepatic vein due to stenosis or ligation; *(b)* partial or incomplete intraoperative hemostasis due to an improper manipulation of the hepatic vein root or trauma to the diaphragm; and (*c*) vascular sutures that could result in a slackening or falling off, an event which usually is due to elevated pressure in the vena cava from patient body movement, such as rolling or coughing intensely [124]. The suspicion of a hemorrhage arises from worsening clinical and laboratory parameters and the presence of blood loss from the abdominal drains [124].

**Figure 5:**
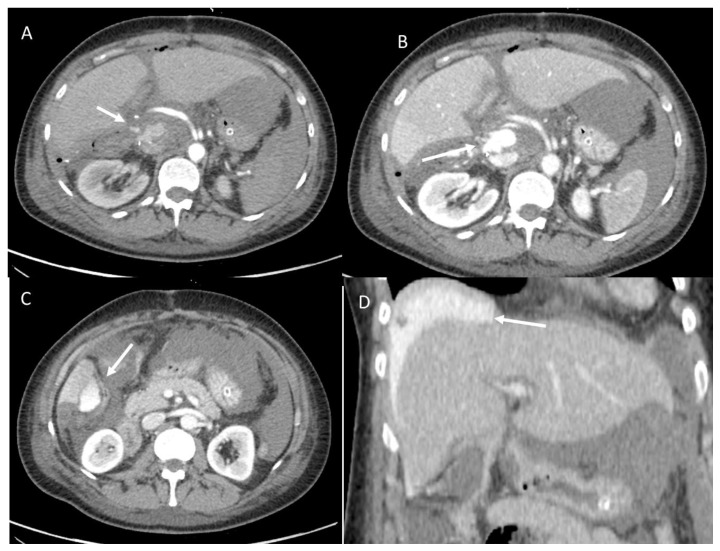
Active bleeding (arrow) during arterial (**A**), portal (**B**), and late (**C**) phase of contrast study. In (**D**), arrow shows contrast collection in perihepatic space.

The US findings should be nonspecific, consisting of a detection of intraabdominal fluid that may be iso-ipo or hyperechogenic or, in selected cases, in color Doppler identification, of turbulent flow at the possible bleeding site [115]. In order to obtain a definite diagnosis of PHH and eliminate other potential causes of bleeding, a multiphasic CT study is mandatory [125]. On baseline examination, a blood collection with a superfluid attenuation of 30–45 HU could be found caudally from the perihepatic space along the right paracolic gutter up to the rectouterine or retro bladder space [125]. A strategy that can help in recognizing the bleeding site is to look for the sentinel clot sign, which is the closest to the origin of bleeding with attenuation values of 45–70 HU [126]. During the arterial phase, the active overflow of contrast material (Figure 5) with a mean attenuation value of 132 HU is evocative of arterial bleeding, which could assume three main morphologic patterns: a focal, spotted, or jet-like appearance [127,128]. The venous phase is certainly diriment in all those cases of low-flow bleeding [128].

#### 3.1.3. Vascular Thrombosis

**Figure 6:**
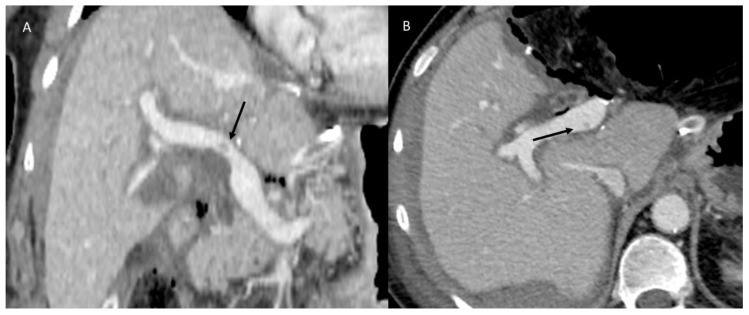
CT portal phase assessment in resected liver metastases patient. The arrow shows (**A**,**B**) mild portal thrombosis.

Postoperative vascular thrombosis is an uncommon complication, which could affect the hepatic and portal branches (Figure 6). A decline in liver function during the early postoperative days is highly indicative of a possible vascular thrombosis. The most frequent event after liver resection is a partial rather than complete hepatic vein occlusion next to resection margins. Although rare, acute Budd–Chiari syndrome (ABCS) may occur after liver resection, with a potentially lethal outcome. Di Domenico et al. described the development of ABCS after an extended right hepatectomy as being due to a contortion of the inferior vena cava or a twist of the left hepatic vein on the remaining liver with an outflow obstruction [129]. The rate of portal vein thrombosis (PVT) is low (about 3%); frequently, a segmental branch is involved (6%) [130]. Clinically, PVT may be undetected because of the absence of specific symptoms. Patients may report abdominal pain if it involves the superior mesenteric vessels and develop bowel congestion or ischemia. In addition, patients could report nausea, vomiting, anorexia, weight loss, diarrhea, or increased abdominal swelling secondary to ascites [131,132]. If acute thrombosis is not identified, collateral vessels will expand, and the patient will advance to cavernous transformation of the portal vein and portal hypertension, which may be evident as varices, splenomegaly, and hemorrhaging [133].

Even rarer is the possibility of arterial thrombosis (HAT), which generally occurs in association with vascular resection and microsurgical reconstruction during the treatment of advanced malignancies [134]. Clinically, HAT can present severely with graft failure (in the case of onset after liver transplant), sepsis, or abscess. In addition, it may present as cholangitis, bile leaks, or modified liver function tests [135,136]. US is a valuable examination tool in suspected vascular thrombosis. A circumscribed thrombus appears as an echogenic area within the affected vessel, with a complete lack or with a slow portal flow in the case of portal vein thrombosis on Doppler images. Color Doppler US is the more appropriate instrument to investigate an ABCS, identified by a loss of triphasic waveforms pattern with a radical decrease in hepatic vein velocity and simultaneous decrease in portal flow, in some cases becoming hepatofugal [137,138]. On the CECT image, during the arterial phase, an intraluminal filling defect referable to a thrombus of the hepatic artery could be easily detected. Venous thrombosis could be intercepted on an unenhanced CT scan as intraluminal hyperattenuating spots within the vessel [115]. Generally, these findings could be associated with a segmental enhancement of the tributary liver parenchyma, paradoxically of increased attenuation due to a compensatory augmentation of the local arterial flow [115].

#### 3.1.4. Biliary Injuries

**Figure 7:**
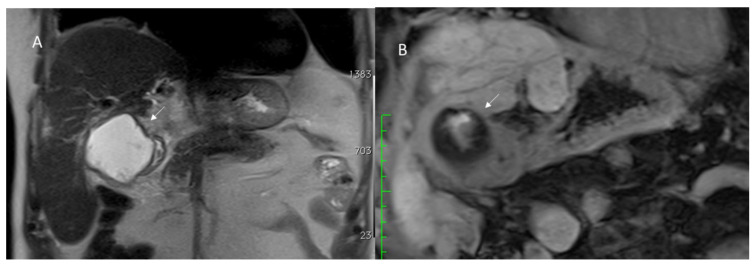
Bile leaks assessed with MRI T2-W sequence (**A**) and hepatospecific phase of contrast study (**B**). Arrow shows leak.

The most frequent postoperative biliary complications are bile leaks (Figure 7), occurring in 5% of cases after liver resection. The ISGLS has suggested a standardized definition of a bile leak, described as a bilirubin level in a drain three times the serum concentration on or after three postoperative days or the necessity of radiologic or operative intervention from a biliary store or bile peritonitis [139]. The leakage can arise from an incompetent bile–digestive anastomosis or from direct damage to the bile ducts during a surgical procedure or removal of a drainage tube [140,141]. If not promptly recognized, a bile leak may lead to sepsis and liver failure with an increased mortality rate [142].

Nagano et al. proposed a classification of postoperative bile leaks after liver surgery in four categories (A-B-C-D), depending on the caliper and site of the injured ductal wall. Specifically, type A identifies self-limiting minor leaks from small bile ducts on the surface of the liver. Type B includes leaks from the main bile duct branches on the liver surface, while type C comprises main duct injuries close to the hepatic hilum. Finally, type D leakage matches with a total transacted duct, without any connection with the main duct [143].

Direct opacification of the bile ducts through the surgical drainage could be appropriate rather than US examination or a CT scan, which could only detect nonspecific collection near the resection margins. Magnetic resonance cholangiopancreatography (MRCP) using gadolinium-based hepatobiliary contrast agents is the gold standard to distinguish the site and the type of the leakage, with a high diagnostic accuracy [144,145].

Invasive diagnostic modalities to define biliary leaks include endoscopic retrograde cholangiopancreatography (ERCP) that allows for the therapeutic management such as the placement of biliary stents and Percutaneous Transhepatic Cholangiography (PTC). ERCP is characterized by some limitations that include the inability to assess the proximal tract of the biliary tree and a difficult passage of the endoscope in postsurgical biliary–enteric anastomosis [146,147]. In the case of postsurgical bile duct damage, interventional radiological treatment includes percutaneous drainage of fluid collections, characterization of the biliary tract anatomy and evaluation of the site and the extent of bile duct injury with PTC, and biliary diversion from the site of bile leakage with external biliary drainage. Percutaneous interventional procedures can arise from definitive treatment or temporization prior to definitive surgical repair that is necessary only in few cases [147,148].

Although extremely rare, an intraoperative diaphragmatic injury may occur, especially during treatment of masses on the right liver. Diaphragmatic injuries generally are self-limiting conditions, but they could be associated with bowel herniation and subsequent perforation. These complications could be hard to classify clinically due to frequent postoperative ileus by aesthetic drugs. CT is often required for a conclusive diagnosis. A diaphragmatic disruption may also lead to biliary fistulae. In this circumstance, CT may show a right pleural diffusion with higher pleural enhancement or a direct passage of a contrast medium agent through the fistulous path [149].

### 3.2. Late Postoperative Complications

#### 3.2.1. Disease Recurrence

Multiphasic CT and MR imaging could be employed in the investigation of disease recurrence.

Gadoxetic acid-enhanced MR imaging is more sensitive than MDCT for discovering the intrahepatic recurrence of HCC after hepatic surgery (98.1% and 67.2%, respectively), with similar specificity values (85% and 90%, respectively) [150].

The CT protocol in postsurgical follow-up varies according to the type of primary resected hepatic tumor. While a baseline scan is unnecessary in most cases, an acquisition during the arterial phase is essential in the evaluation of recurrences from HCC and neuroendocrine tumors [151,152,153] before a portal venous phase. Pre- and postcontrast sequences are mandatory for MRI studies. The detection of biliary dilatation even if there is no obvious mass may always increase the suspicion of recurrence [154,155,156,157].

The rate of recurrence after 5 years from hepatic resection of HCC ranges from 50 to 70% [158,159]. Approximately half of surgically treated cholangiocarcinoma, particularly intrahepatic type, relapse within 5 years after treatment [160]. CT and MRI, including MRCP sequences, are the modalities of choice in the follow-up of these patients, in some cases supplemented by 18FDG positron emission tomography (PET)/CT investigations that can recognize early disease recurrence [161].

**Figure 8:**
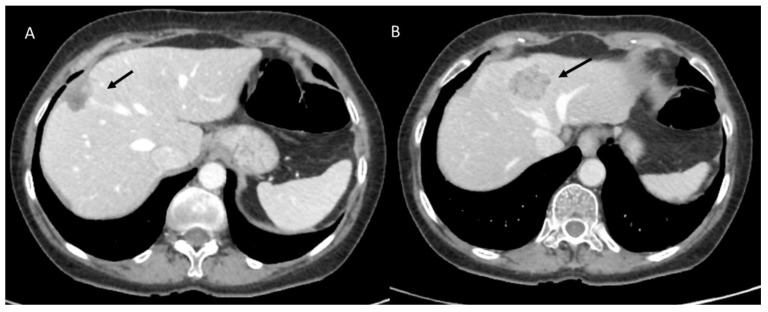
CT assessment in colorectal metastasis-resected patient (**A**). In (**B**), arrow shows a new lesion.

The recurrence rate in patients treated surgically for liver metastases is about 60%, with a particularly high frequency of liver recurrence (40%) [162]. The risk of tumor regrowth along the resection margins is increased if a metastasectomy rather than a segmental resection is performed, due to a higher chance of positive margins [163]. Integrated preoperative planning using hepatospecific contrast-enhanced MRI and CECT is essential to map secondary injuries and assess their link with vascular and biliary structures. In addition, in the postsurgical follow-up, CT, especially the portal phase (Figure 8) and MRI with diffusion and contrast-enhanced sequences, are the methods of choice in identifying disease relapse [150].

#### 3.2.2. Late Strictures and Ischemic Cholangitis

**Figure 9:**
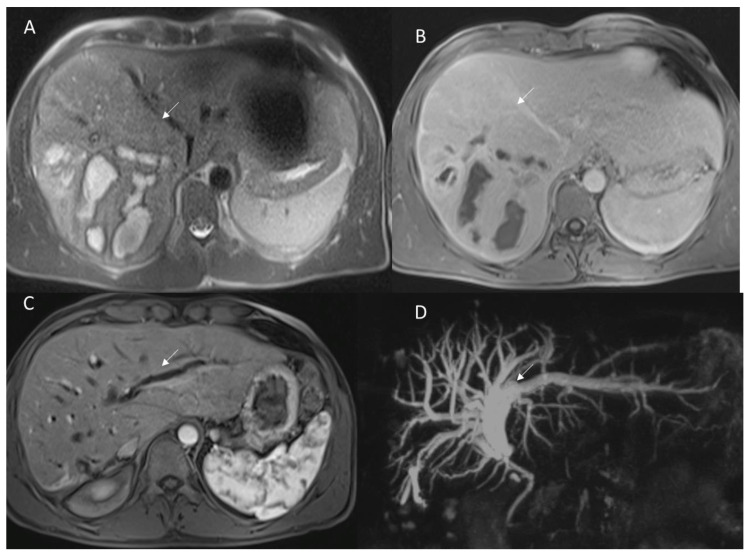
Patient with hepatosarcoma evaluated with MRI study ((**A**): T2-W sequence and (**B**): portal phase of contrast study). At MRI 6-month evaluation of arterial phase (**C**) and cholangiography (**D**) sequences show biliary strictures (arrow).

Biliary strictures (Figure 9) are the most frequent late complications, usually developing anywhere from a few months to many years after surgery [164]. A bile duct is stenotic if the lumen is found sufficiently reduced to justify blood chemistry alterations and impaired bile flow, resulting in obstructive jaundice and liver dysfunction. Despite the reduced caliper, radiological characteristics that may be suggestive of a biliary stricture involve intra- and extrahepatic bile duct dilatation (a diameter of more than 3 or 8 mm, respectively), ductal narrowing, and incomplete display of part of the duct [165]. The most common strictures are the anastomotic type, usually determined by iatrogenic bile duct injury, resulting in bile leakage and scar formation [165].

On CT examination, a dilated fluid-filled Roux-en-Y loop with an upstream dilatation of the biliary tree could be observed, with a fat stranding sign close to the treated area. MRCP is particularly helpful in these kinds of lesions, since the endoscopic approach is only possible in rare cases, and percutaneous transhepatic cholangiography may be related to an increased risk of complications [164]. On MRI images, the obstruction should be considered complete if the morphology of the anastomosis is altered with an empty signal between the ducts and a fluid-filled jejunal loop, in the presence of intrahepatic biliary duct enlargement [164].

Another type of stricture is are the non-anastomotic one, of which ischaemic cholangitis is the most frequent cause. An ischaemic injury resulting from thrombosis of the hepatic artery is the pathophysiological basis for the formation of this type of stenosis. Although, ischaemic cholangitis is a disorder that could occur after orthotopic liver transplantation, it has occurred after liver resection. MRCP is the gold standard technique in the diagnosis of nonanastomotic biliary strictures. The classic picture is that of long ductal segmental hilar stenosis that includes the right and left hepatic ducts and the biliary confluence, connected with the dilatation of the intrahepatic bile tree [141,165].

## 4. Multidisciplinary Assessment

The management after a hepatectomy requires a multidisciplinary treatment, which involves surgeons, interventional radiologists, and gastroenterologists. Although several complications are self-limiting and do not require treatment, when the patient’s life is at risk, it would be appropriate to consider the possibility of a minimally invasive intervention to reduce the risk of further complications in an already critical patient. Therefore, the collaboration of the interventional radiologist and the surgeon should be consistent [166,167,168,169,170]. In addition, a dedicated and expert radiologist would be crucial to identify all critical conditions as soon as possible

## 5. Conclusions

The increase in surgical procedures on the liver has concurrently increased the number of postoperative complications. Elderly patients with significant comorbidities, extended resections, iterative surgeries, and previous chemotherapy are all risk factors for the onset of postoperative complications.

Radiology plays an important role in the early discovery of complications, sometimes with the use of invasive diagnostic modalities, such as ERCP, in their treatment.

Whereas ultrasonography is often the first-line imaging investigation when a postoperative complication is suspected, CT is of greater value for identifying early postoperative pathologic fluid collection, bleeding, and vascular thrombosis, while MRCP is the imaging modality of choice for the characterization of early postoperative bile duct injuries.

MDCT and MR imaging could also be useful in the identification of disease recurrence. Lastly, MRCP also produces the diagnosis of late ischemic cholangitis that may happen after intraoperative arterial injury.

A correct description of these disorders will allow a timely diagnosis and specific management of potentially life-threatening postoperative complications.

## Data Availability

Data are available at link https://zenodo.org/record/6579378#.Yo8zCahBy3A (accessed on 8 May 2022).
